# Psoriasis in skin of color: clinical presentation, diagnostic challenges, and therapeutic considerations^؆^

**DOI:** 10.1016/j.abd.2026.501365

**Published:** 2026-05-18

**Authors:** Anderson Costa, Ricardo Romiti

**Affiliations:** a Department of Dermatology, Hospital Ipiranga, São Paulo, SP, Brazil; b Department of Dermatology, Faculty of Medicine, Universidade de São Paulo, São Paulo, SP, Brazil

**Keywords:** Biological products, Ethnic groups, Healthcare disparities, Psoriasis, Skin pigmentation

## Abstract

Psoriasis in individuals with Skin of Color (SOC) shows distinctive clinical features and particularities that require careful consideration for accurate recognition and care. Erythema is often less apparent, and plaques may appear violaceous or dark brown. These features, together with limited discussion of these nuances in textbooks and articles, contribute to delayed diagnosis, greater reliance on skin biopsy, and reduced diagnostic accuracy. Although overall prevalence may be lower than in White populations, SOC patients frequently present with greater body-surface involvement, thicker scale, and a disproportionate impact on quality of life. Early, effective control is critical not only to limit systemic comorbidities and quality of life impairment but also to prevent post-inflammatory dyspigmentation, which can persist for years after clinical resolution and substantially affect psychosocial well-being. Despite major advances in psoriasis research, important gaps remain regarding genetic drivers, immunopathogenesis, and the comparative effectiveness and safety of therapies across diverse populations, gaps compounded by the persistent underrepresentation of SOC in clinical trials. This review synthesizes current evidence on epidemiology, clinical presentation, diagnostic pitfalls, pigmentary sequelae, psychosocial dimensions, and therapeutic options so as to support equitable, patient-centred outcomes for SOC patients living with psoriasis.

## Introduction

Psoriasis is a chronic, immune-mediated systemic inflammatory disease that affects the skin and joints and is associated with multiple comorbidities.^1^ It carries a substantial quality-of-life and psychological burden, particularly among people with Skin of Color (SOC), where the disease may be more severe and stigmatizing.^2^

Although prevalence varies across ethnic groups and regions, most epidemiologic data come from White populations, leaving important gaps for SOC.^3^, ^4^ Emerging evidence suggests that while plaque psoriasis may be less prevalent in SOC, it often involves greater body-surface area and yields a higher quality-of-life impact.^4^

Clinical presentation also differs in SOC: plaques are frequently violaceous or hyperpigmented, erythema may be subtle, and resolution commonly leaves persistent post-inflammatory dyschromias, which many patients find more distressing than active lesions.^5^

Despite therapeutic advances, important disparities persist in the diagnosis, treatment access, and outcomes of psoriasis in SOC populations. Structural and socioeconomic barriers, implicit bias in healthcare delivery, underrepresentation in clinical trials, and the lack of culturally sensitive approaches all contribute to suboptimal care.^6^ Recognizing these differences is essential to deliver equitable and effective care.

## Methods

This narrative review was conducted through a comprehensive literature search of the PubMed/MEDLINE, Embase, and Cochrane Library databases, covering articles published between 2000 and 2025. The search strategy utilized MeSH terms and keywords including “Psoriasis”, “Skin of Color”, “Black Skin”, “darker skin phototypes”, “Fitzpatrick types IV‒VI”, “ethnic skin”, “pigmented skin”, “African”, “Asian”, “Hispanic”, combined with terms related to clinical presentation, diagnosis, treatment, biologics, and health disparities.

Articles were selected based on clinical relevance, focusing on studies that included diverse racial and ethnic populations to synthesize current evidence on diagnostic and therapeutic nuances in SOC (Table 1).

**Table 1:** Summary of key points ‒ psoriasis in SOC.

Unmet Needs in Care	Diagnostic Challenges	Treatment Challenges
Lower healthcare utilization rates, fewer physician visits, and higher hospitalization rates due to economic burdens and lack of culturally competent care	Diagnostic delays in SOC patients are 3× longer compared to lighter-skinned populations	Underrepresentation in clinical trials: Only 15.7% of psoriasis clinical trial participants are non-white
Psoriasis has a greater negative impact on quality of life in SOC populations compared to white populations	Up to 4× more biopsies required for diagnosis due to lower accuracy of visual diagnosis alone	Cultural preferences and aversion to tanning limit acceptance and adherence to phototherapy among Asian patients
Significant underrepresentation in dermatology educational resources: only 4.5% of images in dermatology textbooks depict darker skin types	Difficulty recognizing erythema leading to underestimated severity and artificially low PASI scores; greater initial body surface area involvement	Preference for topical therapies that minimize risk of hypopigmentation and are compatible with hair-care practices in African American patients
Delay in initial presentation to dermatologists due to reliance on traditional treatments before medical consultation	Frequent confusion with other skin conditions (e.g., lichen planus, lupus), leading to misdiagnosis and inappropriate treatments	Genetic polymorphisms influencing Methotrexate metabolism; higher risk of hypertension and renal complications with Cyclosporine in African American patients

## Terminology: Why use the term “skin of color”

In the global dermatology literature, the term SOC has gained prominence to describe populations with richly pigmented skin. However, in Brazil, there is no universally accepted term to refer to the non-White population within medical or dermatological contexts. Expressions such as *ethnic skin* have appeared in texts but are increasingly viewed as problematic due to their Eurocentric bias.^7^

The use of the term *ethnic skin* often implicitly positions White skin as the default, thereby marginalizing the broad phenotypic diversity of global populations.^8^ Consequently, several authors and international guidelines recommend abandoning terms such as *ethnic skin*, *Hispanic skin*, and *Asian skin* in favor of SOC ‒ a term that more accurately captures biologically relevant characteristics in dermatological care while avoiding cultural reductionism.^9^

In this article, the authors have opted to adopt the term SOC, as it aligns with current international discourse, facilitates communication with the global scientific community, and acknowledges the heterogeneity of the Brazilian population. Nevertheless, the authors recognize that this terminology is not without limitations and should be continuously refined to better reflect the specific dermatological needs of diverse populations.

## Skin of color in the Brazilian context

**Figure 1:**
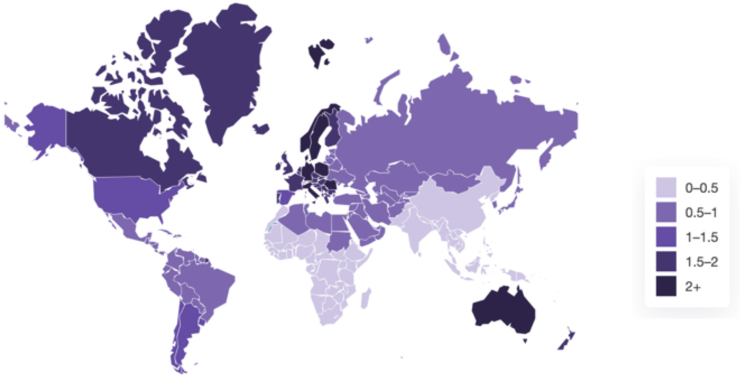
Global prevalence of psoriasis – Global Psoriasis Atlas. Global Psoriasis Atlas. Global prevalence of psoriasis – heatmap [Internet]. Manchester: International Psoriasis Council; c2025 [cited 2025 Jun 19]. Available from: https://www.globalpsoriasisatlas.org/en/explore/prevalence-heatmap.

Brazil is one of the most genetically diverse countries in the world, a consequence of centuries of admixture between Indigenous peoples, enslaved Africans, and European settlers, along with more recent immigration from various global regions.^10^ This complex genetic mosaic has led to a wide spectrum of skin tones across the population, which presents both diagnostic and therapeutic challenges in dermatological practice (Fig. 1).

The Brazilian Institute of Geography and Statistics (IBGE) classifies the population into five self-reported racial/ethnic categories: *branca* (White), *preta* (Black), *parda* (mixed-race), *indígena* (Indigenous), and *amarela* (East Asian). This classification system is based on self-identification and reflects the country’s sociocultural and historical dynamics.^11^ In academic and policy contexts, the term “negro” is commonly used to collectively refer to individuals who identify as *preto* or *pardo*, acknowledging shared experiences and the structural disadvantages historically faced by Afro-descendant populations.

According to the 2022 Census, 20.7 million Brazilians (10.2%) identified as *preto* and 89.2 million (43.5%) as *pardo*. Combined, these groups form a population of 109.9 million people, representing over 50% of the total population.^12^ This demographic reality marked the first time in Brazilian census history that the *negra* population became the majority.

This underscores the need to recognize and address disparities in dermatological care. Clinical manifestations of skin diseases ‒ such as psoriasis ‒ can vary significantly according to skin tone, and failure to account for these differences may lead to underdiagnosis or delayed treatment.^4^ Tailored diagnostic strategies and inclusive therapeutic approaches are essential to ensure equitable and effective care for all population groups.

## Epidemiology

**Figure 2:**
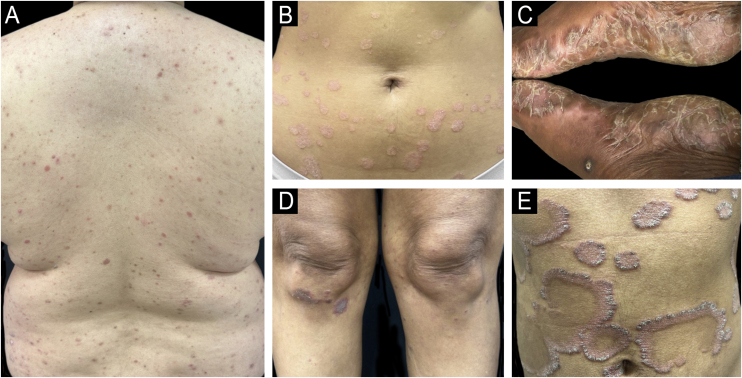
Clinical variability of psoriasis across different skin phototypes. (A) Small-plaque psoriasis in a patient with phototype III ‒ a variant more frequently observed in Asian populations, characterized by numerous small lesions rather than classic large plaques. (B) Classic plaque psoriasis in a patient with skin phototype III, showing well-demarcated erythematous plaques with silvery-white scale. (C) Palmoplantar psoriasis in a patient with skin phototype VI. Marked hyperkeratosis and yellowish scale on the soles may mimic chronic eczema or tinea. (D) Psoriasis in a patient with skin phototype V. Violaceous papules and plaques with gray scaling are observed on typical extensor surfaces. (E) Annular psoriasis in a patient with skin phototype IV, with well-defined arcuate and serpiginous plaques exhibiting central clearing.

Psoriasis affects men and women equally, with a mean age of onset around 33-years.^13^ Its prevalence varies significantly across geographic regions and ethnic groups. Globally, approximately 125 million individuals are estimated to live with the disease (Fig. 2).^1^

The condition is notably more prevalent among individuals of European ancestry, particularly in northern European countries such as those in Scandinavia, where prevalence rates can reach up to 5%.^13^ In contrast, psoriasis appears to be rare or even absent among some Indigenous populations. A dermatologic survey involving 25,000 individuals from Andean communities reported no cases of psoriasis, reinforcing this observation.^14^, ^15^

Similarly, a population-based clinical survey conducted in an isolated indigenous community in the Yanomami Territory in Northern Brazil found no documented cases of psoriasis among 555 individuals,^16^ suggesting the potential influence of protective environmental or genetic factors. In Asia, prevalence also shows considerable variability. While Malaysia reports higher rates (4%–5%), countries like China, Japan, and Sri Lanka exhibit much lower prevalence, ranging between 0.05% and 0.47%.^4^

In the United States, recent population-based studies have reported psoriasis prevalence rates of 3.6% among White individuals, 1.9% among African Americans, and 1.6% among Hispanic/Latino populations.^15^ Data from the NHANES 2009–2010 survey corroborate this ethnic gradient, with African Americans estimated to have a 52% lower prevalence than White Americans, depending on the methodology used, with reported rates ranging from 0.7% to 1.9%, depending on study methodology.^17^

Interestingly, the prevalence in African Americans mirrors that seen in several West African countries, including Nigeria, Mali, and Senegal, where estimates range from 0.3% to 0.8%.^14^ This similarity is hypothesized to reflect shared ancestral origins, as the majority of African Americans trace their lineage to West Africa.^18^

In Brazil, the overall prevalence of psoriasis is estimated at 1.31%, based on a population-based telephone survey conducted in all 26 state capitals between October 2015 and January 2016.^19^ Regional differences were statistically significant (p = 0.02), with higher prevalence in the South and Southeast, and lower rates in the North and Northeast. Although there are no national epidemiological data stratified by race or skin color, this geographic gradient appears to reflect Brazil’s ethnic distribution: the North and Northeast regions have a greater proportion of individuals of African and Indigenous ancestry. In contrast, the South and Southeast regions, with the highest reported psoriasis prevalence, have larger proportions of individuals of European ancestry.^12^

In addition to possible genetic factors, other explanations for this disparity include differences in Ultraviolet (UV) exposure ‒ given the higher solar irradiance in northern Brazil, which may exert a protective effect ‒ and unequal access to dermatologic care.^19^ Interestingly, these findings echo U.S. data showing lower psoriasis prevalence among African Americans compared to White individuals and may suggest that individuals with African ancestry have a lower genetic predisposition to develop psoriasis, although further studies are needed to confirm this hypothesis.^15^, ^18^

Globally, a critical barrier to understanding psoriasis epidemiology in SOC populations is the lack of population-based data. A recent analysis highlighted that only 19% of countries worldwide currently have published epidemiological data on psoriasis.^20^ This data gap is particularly prominent in Africa, Asia, and Latin America, where many studies are limited to small hospital-based cohorts with variable diagnostic criteria.^3^

Recognizing this need, the Global Psoriasis Atlas (https://www.globalpsoriasisatlas.org), a collaborative initiative involving the International Federation of Psoriasis Associations, the International League of Dermatological Societies, and the International Psoriasis Council, has been working to address these disparities and improve the understanding of psoriasis epidemiology in underrepresented populations.

## Genetic insights

Psoriasis is a complex, immune-mediated disease with significant genetic involvement, with heritability estimates exceeding 60% across various populations.^21^ Twin and family-based studies underscore this genetic component, showing higher concordance rates among monozygotic twins compared to dizygotic twins.^22^ Genome-Wide Association Studies (GWAS) have significantly advanced our understanding of psoriasis genetics, identifying over 80 susceptibility loci across diverse ethnicities.^13^

The PSORS1 locus, located within the Major Histocompatibility Complex (MHC) on chromosome 6p21, remains the primary genetic risk factor,^15^ within this region, the HLA-C06:02 allele is strongly linked to early-onset and guttate psoriasis.^4^ Nevertheless, HLA-C06:02 frequency varies significantly across ethnicities, being most common among Europeans (up to 70% of psoriasis cases) and notably lower in Asian and African populations.^4^

In contrast, HLA-C01:02 (Cw1) is more prevalent in certain Asian populations, including Southern Chinese, Thai, and Japanese patients, and associates predominantly with severe forms like erythrodermic and pustular psoriasis, later disease onset, and poorer response to conventional treatments. Additional alleles such as HLA-B46 and HLA-A02:07 have also been implicated specifically within Asian cohorts, further highlighting genetic diversity among populations.^23^

Beyond the HLA region, numerous non-HLA genes linked to innate and adaptive immunity are also implicated in psoriasis. Notably, the IL-23/Th17 axis genes, including IL23R, IL12B, TYK2, STAT3, and TRAF3IP2, exhibit strong associations across various ethnic groups.^13^, ^21^ Moreover, variants in NF-κB signaling genes like CARD14 and TNFAIP3 have particular relevance for Asian and Middle Eastern populations.^21^

African populations display genetic heterogeneity, with paradoxically low psoriasis prevalence despite higher frequencies of some susceptibility alleles, suggesting protective genetic or environmental modifiers.^4^

In the Brazilian context, genetic admixture creates a unique genomic landscape that could impact psoriasis susceptibility, clinical presentation, and therapeutic responses. Recent genomic studies highlight significant regional variations in ancestry across Brazil, underscoring the need for tailored genetic research and healthcare strategies that reflect this diversity.^10^

## Pathophysiology

Psoriasis is characterized by complex interactions between genetic susceptibility, environmental triggers, and immune dysregulation.^1^ The pathogenesis of plaque psoriasis, the most common clinical variant, is primarily driven by a feed-forward inflammatory loop centered on the TNF-α/IL-23/IL-17 axis.^24^

This cascade begins with activation of innate immune cells such as plasmacytoid dendritic cells and macrophages. These cells produce proinflammatory cytokines, including Interferon-Alpha (IFN-α), Interleukin-1 Beta (IL-1β), and Tumor Necrosis Factor-Alpha (TNF-α), which together promote the activation and maturation of myeloid dendritic cells. IL-12 promotes Th1 differentiation, while IL-23 plays a critical role in the survival and expansion of Th17 and Th22 cells.^1^ The Th17 pathway is now recognized as the principal driver of psoriatic inflammation, with IL-17 and IL-22 being key cytokines leading to keratinocyte hyperproliferation, impaired differentiation, and the recruitment of additional immune cells to the skin.^24^

Keratinocytes are not passive bystanders but active participants in psoriasis pathogenesis. They respond to inflammatory cytokines by producing chemokines, antimicrobial peptides, and further inflammatory mediators, thereby amplifying the immune response. In addition, dysregulation of skin barrier function, with alterations in genes like those from the Late Cornified Envelope (LCE) cluster, may further exacerbate the inflammatory loop.^1^, ^13^

Although data on the pathophysiological mechanisms of psoriasis in SOC populations are still limited,^5^ some studies suggest possible differences in skin barrier function and immune response across racial and ethnic groups.^6^ In an experimental model using 3D human skin equivalents from African American and White Non-Hispanic keratinocytes, African American cells showed higher baseline pro-inflammatory gene expression and an amplified response to TNF-α stimulation.^25^ However, these findings have yet to be directly correlated with clinical differences in disease severity or treatment outcomes.

Clinically, patients with SOC often exhibit distinctive psoriatic lesion morphology and a greater tendency toward post-inflammatory pigmentary changes, which may reflect underlying pathophysiological differences.^4^ However, current evidence does not support the presence of fundamentally distinct immunopathogenic pathways when compared to White populations.^6^

## Clinical presentation in SOC and diagnostic challenges

**Figure 3:**
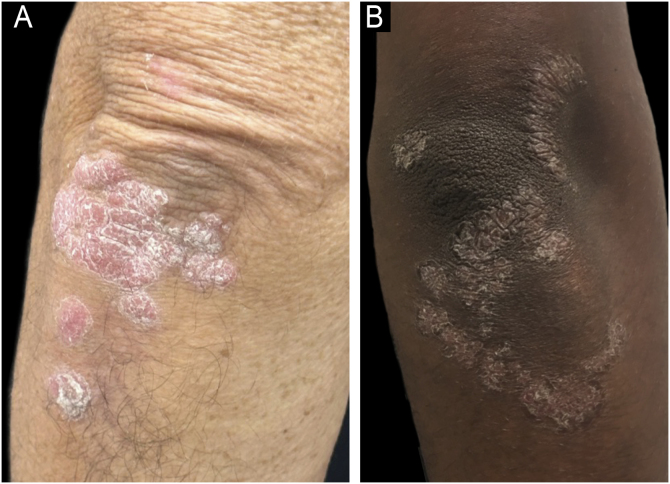
Clinical presentation of psoriasis plaques demonstrating the challenges of erythema recognition in SOC. (A) A typical erythematous plaque of psoriasis clearly seen on lighter skin. (B) A psoriasis plaque presenting with violaceous, hyperchromic, and grayish tones on darker skin, illustrating the variability in visual appearance. Such differences underscore the importance of recognizing non-traditional signs of inflammation to accurately diagnose and assess psoriasis severity in SOC patients.

The clinical presentation of psoriasis varies considerably depending on the patient's skin phototype, posing particular diagnostic challenges in individuals with SOC. While plaque psoriasis is classically described in fair-skinned populations (Fitzpatrick phototypes I–III) as erythematous plaques covered by silvery-white scales, these typical presentations are often substantially modified in patients with darker skin tones (phototypes IV–VI), leading to diagnostic delays and errors (Fig. 3).^2^, ^4^

One major challenge in diagnosing psoriasis in SOC populations is the reduced visibility of erythema. Rather than presenting with classic red or salmon-colored plaques, lesions in darker skin tones often manifest as violaceous, dark brown, hyperpigmented, or grayish tan-colored patches due to masking of underlying inflammation by melanin.^26^ This altered appearance significantly decreases visual diagnostic accuracy, contributing to frequent misdiagnosis or delayed diagnosis.^2^

A large-scale visual diagnostic challenge found diagnostic accuracy to be significantly lower in darker skin compared to lighter skin tones, confirming the impact of underrepresentation and inadequate training in dermatologic education.^27^, ^28^ The use of the term “erythema” itself has been recently challenged, given its limited applicability and imprecise nature in describing inflammation in darker skin phototypes, and experts have recommended reevaluating or replacing this descriptor to improve clinical accuracy and inclusivity.^29^

As a consequence, patients with darker skin experience notably longer diagnostic delays, often triple the time^2^ taken for lighter-skinned counterparts, and require significantly more diagnostic biopsies. Indeed, studies have demonstrated that the probability of biopsy utilization to confirm a psoriasis diagnosis is four times greater in SOC individuals.^2^

Diagnostic tests such as Brocq's methodical curettage, Auspitz sign, and the candle wax sign remain particularly useful in SOC, as these physical findings do not depend on the visibility of erythema and therefore help confirm the diagnosis.

In addition, patients with SOC commonly present with thicker plaques, more pronounced scaling, and a higher degree of body surface area involvement. Specifically, palmoplantar psoriasis and severe plaque psoriasis involving more than 10% of body surface area are disproportionately prevalent among Black and Hispanic populations compared to White populations.^2^, ^4^

**Figure 4:**
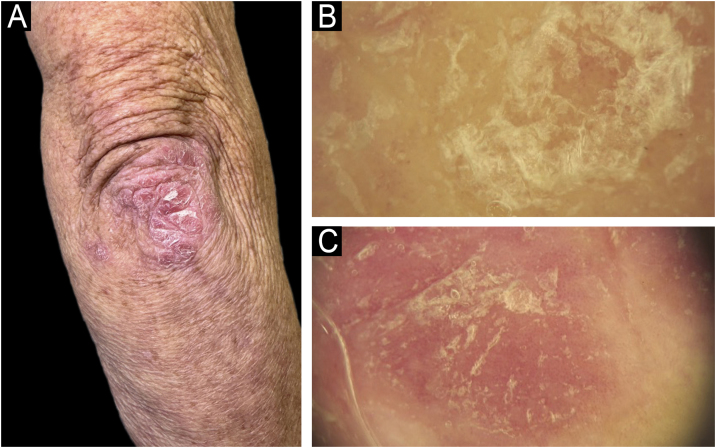
Dermoscopy of plaque psoriasis. (A) Clinical image of a psoriatic plaque on the elbow, showing well-demarcated erythematous plaque with silvery scale. (B) Dermoscopic image highlighting white scales and dotted vessels. (C) Dermoscopy in a lighter phototype showing prominent regular dotted vessels on a homogeneously erythematous background with white scaling ‒ classic findings in psoriasis.

Dermoscopy is increasingly recognized as a valuable adjunctive diagnostic tool, especially important in SOC populations. Typical dermoscopic features of psoriasis: regular dotted vessels, homogeneous background erythema (when visible), and scales remain consistent irrespective of skin tone. Dermoscopy significantly enhances diagnostic accuracy by enabling visualization of subtle vascular changes and distinguishing psoriasis from other papulosquamous diseases, thereby reducing unnecessary invasive procedures (Fig. 4).^30^

**Figure 5:**
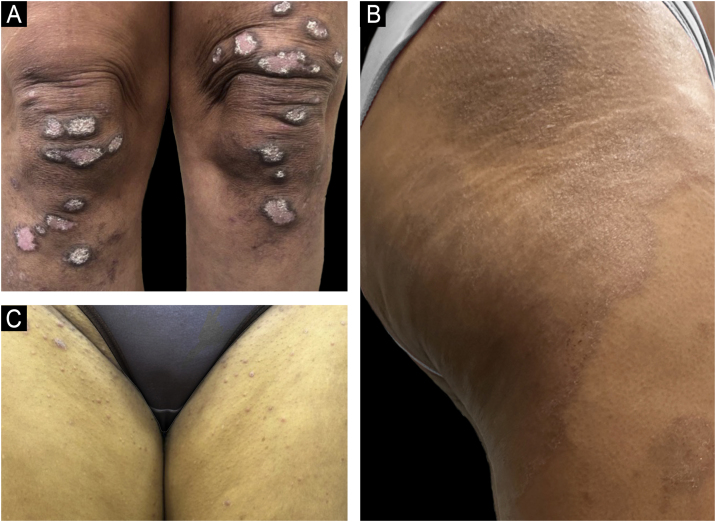
Diagnostic challenges in SOC. (A) Patient with chronic cutaneous lupus erythematosus presenting with discoid lesions. (B) Patient with psoriasis previously misdiagnosed as lichen planus for several years. (C) Patient with atopic dermatitis without visible erythema, a common presentation in darker skin tones.

A further diagnostic consideration in SOC patients is the spectrum of differential diagnoses, which notably includes conditions such as lichen planus, sarcoidosis, cutaneous lupus erythematosus, tinea corporis, secondary syphilis, eczema, and mycosis fungoides. These conditions frequently exhibit morphological overlap with psoriasis in darker skin, complicating clinical differentiation (Fig. 5).^2^, ^4^

Asian populations frequently present a particular variant termed small plaque psoriasis, characterized by stable, chronic lesions typically smaller than 2 cm, contrasting with the larger plaques more common in Western populations. Despite their smaller size and milder clinical appearance, molecular studies have demonstrated a similar activation of the IL-17 pathway in these small plaques.^31^

Given these clinical complexities, it is essential to recognize the profound diagnostic challenges faced by dermatologists treating SOC patients. Educational resources, clinical guidelines, and research studies remain insufficiently representative of darker skin types, directly influencing clinicians' diagnostic accuracy and management strategies.

A recent analysis of dermatology textbooks revealed that psoriasis images in SOC accounted for only 7.3%^32^ of all psoriasis-related photographs, despite darker-skinned populations constituting a significant and growing proportion of global demographics.

**Figure 6:**
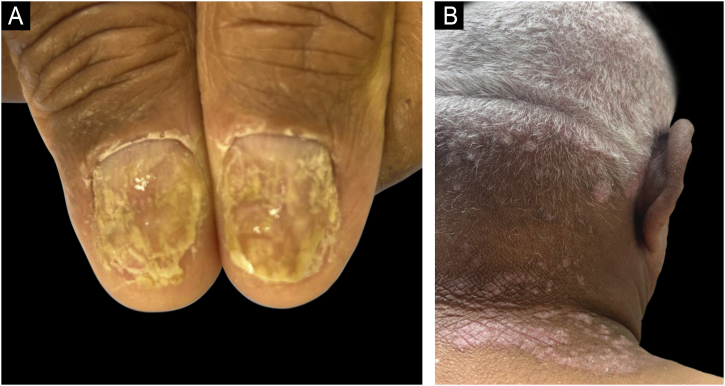
Psoriasis manifestations in hard-to-treat areas. (A) Nail involvement in a patient with Fitzpatrick phototype VI, showing subungual hyperkeratosis, onycholysis, and oil drop sign. (B) Scalp involvement in the same patient, a site commonly affected in patients with skin of color and often associated with increased disease burden.

Enhancing dermatologic training, updating clinical resources, and improving representation in research are imperative to reduce existing racial and ethnic disparities and significantly improve patient outcomes in SOC populations (Fig. 6).

## Dyschromias in psoriasis in skin of color

**Figure 7:**
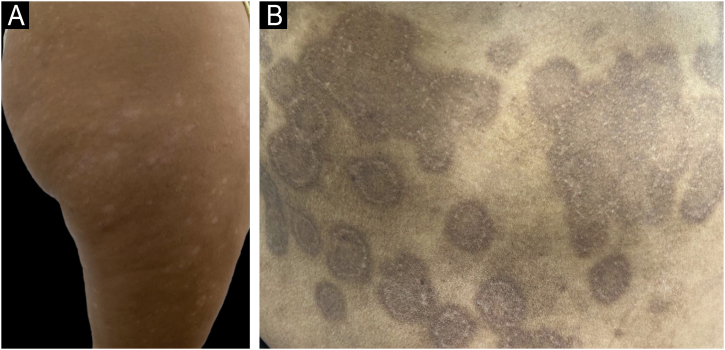
Post-inflammatory dyschromias. (A) Hypopigmented macules on the thigh of a phototype IV patient. (B) Hyperpigmented macules with annular configuration on the trunk of a phototype V patient. Persistent dyschromias represent a significant concern for SOC patients, often impacting quality of life more severely than the initial inflammatory lesions themselves.

Post-inflammatory dyschromias, encompassing both hyperpigmentation and hypopigmentation, represent a prominent clinical challenge in managing psoriasis in patients with SOC. These pigmentary changes frequently persist after inflammatory lesions resolve and often impact patient quality of life more profoundly than active psoriasis itself, due to their highly visible and long-lasting nature, affecting patient self-esteem and social interactions (Fig. 7).^4^, ^26^

Post-inflammatory Hyperpigmentation (PIH) is particularly common in patients with darker skin phototypes. It arises secondary to inflammatory mediators such as IL-1, IL-6, IL-18, and prostaglandins (notably PGE2 and PGF2α), which directly stimulate melanogenesis. These mediators upregulate the expression of tyrosinase and other melanogenic enzymes, enhancing melanin synthesis and subsequent epidermal pigmentation following inflammation.^33^

Hypopigmentation also occurs as a distinct post-inflammatory sequela of psoriasis. The most classic and specific manifestation of hypopigmentation associated with psoriasis is the Woronoff ring, a pale annular zone appearing around healing psoriasis plaques.^34^ Described over a century ago, the Woronoff ring results from cytokine-mediated disruption of melanogenesis during inflammation.

Recent studies have clarified the underlying mechanism, implicating IL-17 and TNF-α in simultaneously suppressing melanin production while paradoxically increasing melanocyte proliferation within these lesions. This imbalance leads to visibly decreased pigmentation despite an increased density of melanocytes, forming the characteristic depigmented halo around regressing plaques.^34^

In addition to the Woronoff ring, post-inflammatory hypopigmentation areas following psoriasis can occur, often exacerbated by therapeutic interventions such as phototherapy and topical corticosteroids. These treatment modalities, while effective for psoriasis management, may further disrupt melanocyte function or melanin distribution, compounding hypopigmentation issues and highlighting the careful balance needed in therapeutic decisions.^34^

Depth of pigment also matters clinically: epidermal PIH typically appears tan-to-brown and can persist for months, whereas dermal PIH shows a blue-gray hue and may be long-lasting or permanent.^35^ Because ultraviolet exposure and ongoing inflammation aggravate PIH, rigorous photoprotection and early control of cutaneous inflammation are foundational. In fact, contemporary PIH care frameworks emphasize that the first step is to treat the underlying inflammatory disease promptly; although that algorithm is often illustrated with acne,^36^ the same principle should be applied in psoriasis to minimize deeper, harder-to-treat pigment deposition.^37^

Therapeutic options for PIH are limited and include topical retinoids, azelaic acid, hydroquinone, and ‒ more recently ‒ thiamidol, a selective inhibitor of human tyrosinase, as well as other available lightening agents and chemical peels. Energy-based devices (e.g., 1064-nm Q-switched Nd:YAG) can be useful in expert hands but warrant caution in richly pigmented skin. A practical barrier for extensive body involvement is that most depigmenting topicals are sold in small volumes and may be cost-prohibitive for large surface areas. This limitation should be discussed during shared decision-making, prioritizing sites that most affect patient well-being.^37^

Tranexamic Acid (TXA) is an increasingly discussed adjunct for PIH. Mechanistically, TXA can reduce UV-induced melanogenesis and dampen pro-pigmentary signaling (e.g., via effects on arachidonic-acid metabolites and autophagy). Small, randomized studies around laser procedures show mixed results for prevention but suggest faster PIH resolution when TXA is continued post-procedure. Safety data in nonsurgical patients without thrombotic risk factors are reassuring, though standard contraindications and risk counseling remain essential.^38^ Beyond pigment specifically, preclinical work suggests topical TXA may also attenuate psoriasis-like inflammation by blunting IL-17-driven NF-κB signaling and inflammasome activation ‒ raising the possibility that TXA could, in carefully selected cases, both improve pigmentary outcomes and assist in controlling psoriatic inflammation.^39^

Despite the recognized significance of these pigmentary changes, they remain understudied, and current dermatological literature inadequately addresses therapeutic management specifically tailored for SOC populations. Expanding clinical studies and refining management strategies focused on pigmentary sequelae are, therefore, critical steps toward improving outcomes and quality of life for patients with psoriasis.

## Psoriatic arthritis

Psoriatic Arthritis (PsA) is a chronic inflammatory arthritis associated with psoriasis, affecting up to 30% of psoriasis patients, typically developing within 7- to 10-years after the onset of cutaneous symptoms. However, joint involvement may precede skin lesions in approximately 15% of patients or occur simultaneously.^1^

PsA presents heterogeneously, characterized by peripheral arthritis, axial involvement, dactylitis, enthesitis, and nail disease, significantly affecting quality of life and leading to functional impairment if untreated. Diagnosis is clinical, supported by imaging and classification criteria such as the CASPAR (Classification Criteria for Psoriatic Arthritis). Common presenting symptoms include joint pain, swelling, stiffness, fatigue, and functional limitation, typically worsened in the morning or after prolonged rest.^1^, ^13^

Despite the broad epidemiological knowledge regarding PsA, data specifically examining the clinical presentation and epidemiology among SOC populations remain limited. Recent studies indicate significant racial and ethnic disparities in PsA phenotypes, disease activity, and clinical outcomes. Specifically, Hispanic and non-white individuals with PsA are more likely to exhibit higher tender joint counts, elevated patient-reported disease activity scores (e.g., RAPID3), and increased prevalence of moderate to severe cutaneous psoriasis despite similar usage of systemic therapies. Radiographic axial involvement, traditionally associated predominantly with Caucasian populations due to the association with HLA-B27, has been increasingly documented among non-white individuals, highlighting the phenotypic diversity of PsA across different ethnicities.^40^

Furthermore, PsA patients from SOC populations frequently experience diagnostic delays and increased morbidity, partially attributable to underrecognition and underrepresentation in clinical studies and medical education resources. These patients often present with higher disease burden and worse patient-reported outcomes at initial clinical visits, reflecting potential disparities in healthcare access and socioeconomic barriers.^4^

Additionally, studies reveal that Asian populations, particularly South Asians, may exhibit distinct PsA phenotypes characterized by a younger age of onset, more severe joint symptoms, and increased frequency of enthesitis and dactylitis compared to their Caucasian counterparts. These clinical differences underscore the necessity for increased clinician awareness, culturally competent healthcare strategies, and inclusion of diverse populations in clinical trials.^15^, ^40^

## Comorbidities in psoriasis patients with skin of color

Psoriasis is recognized as a systemic inflammatory disease, frequently associated with various comorbidities beyond joint involvement. Commonly reported comorbidities among psoriasis patients include cardiovascular diseases, metabolic syndrome, obesity, diabetes mellitus, hypertension, hyperlipidemia, chronic kidney disease, and psychiatric disorders such as depression and anxiety. The presence of these conditions significantly influences patient management strategies and long-term prognosis, underscoring the importance of comprehensive clinical assessment beyond cutaneous manifestations.^1^

Although these comorbidities are well-established in psoriasis populations generally, research specifically examining the comorbidity burden in SOC patients remains relatively limited. Recent studies utilizing large-scale, population-based databases, such as the National Ambulatory Medical Care Survey (NAMCS), have provided valuable insights into racial and ethnic differences. Interestingly, a recent comprehensive analysis comparing the comorbidity burden in SOC psoriasis patients to White psoriasis patients, measured by the Charlson Comorbidity Index (CCI), found no statistically significant differences among racial groups. Hypertension, arthritis, hyperlipidemia, and diabetes were among the most frequently reported conditions across all races, suggesting commonalities rather than differences driven by biological or genetic factors associated with race alone.^41^

However, disparities in health outcomes, treatment access, and quality of life persist among SOC patients, largely attributable to socioeconomic status, healthcare access, and potential underrepresentation in clinical guidelines and research. Despite similar comorbidity burdens between SOC and White populations, SOC patients frequently experience worse disease outcomes due to delayed diagnosis, misdiagnosis, and inadequate management arising from systemic inequities. Therefore, addressing these disparities requires targeted interventions focused on improving healthcare accessibility.^41^

## Special considerations in the treatment of psoriasis in skin of color

### Topical therapies

Topical therapies remain first-line treatments for mild psoriasis across all skin types. However, treatment of psoriasis in SOC populations demands careful consideration due to increased risk of post-inflammatory hypo- and hyperpigmentation. The prolonged use of potent topical corticosteroids is often discouraged among patients with darker skin due to their potential for causing significant hypopigmentation, atrophy, and striae, which negatively impact patient adherence.^42^

Non-bleaching alternatives, such as vitamin D analogs (e.g., calcipotriene) and calcineurin inhibitors (e.g., tacrolimus and pimecrolimus), are often preferred, particularly for facial and intertriginous areas.^4^ A recent analysis highlighted the efficacy and safety of calcipotriene/betamethasone dipropionate foam in SOC patients, demonstrating good clinical outcomes with an acceptable pigmentary safety profile.^43^

When managing scalp psoriasis in SOC patients, particularly those of African descent, clinicians should tailor topical regimens to accommodate cultural hair care practices and styling preferences. Daily washing with medicated shampoos, often recommended in standard protocols, may not be feasible for many African American women due to concerns over hair dryness, breakage, and disruption of protective hairstyles such as braids, weaves, and locks.^15^

Instead, a more suitable approach involves weekly use of medicated shampoos combined with once- or twice-daily application of corticosteroids in vehicles compatible with the patient's hair texture and styling habits. Oil-based preparations, lotions, and emollient foams are often better accepted. Engaging in a brief discussion about vehicle preferences, hair care routines, and potential use of traditional scalp treatments is essential to optimize adherence and treatment satisfaction in this population.^15^

### Phototherapy

Phototherapy remains a cornerstone in the management of moderate-to-severe psoriasis, particularly in patients with more than 10% body surface area involvement or in those refractory to topical therapies. Common phototherapeutic modalities include broadband Ultraviolet B (BB-UVB), Narrowband UVB (NB-UVB), and Psoralen Plus UVA (PUVA), each with unique efficacy and safety profiles.

BB-UVB, which utilizes the entire UVB spectrum (254–313 nm), has demonstrated efficacy but has largely been supplanted by NB-UVB (311–313 nm) due to superior clearance rates and a better safety profile in head-to-head trials. PUVA therapy, combining UVA (320–400 nm) with psoralens, has shown high efficacy, with clearance rates approaching 89% in some studies.^44^ However, concerns about long-term carcinogenic risks, especially squamous cell carcinoma, have limited its use.^45^ Additionally, in SOC populations, PUVA is associated with a higher risk of dyschromias, making NB-UVB generally the preferred modality.^44^

An important consideration in SOC patients is the impact of increased melanin content on UV dose calculation and treatment tolerability. Traditionally, clinicians have escalated NB-UVB doses in darker-skinned individuals to achieve mild erythema (erythemogenic threshold), assuming it correlates with treatment efficacy. However, emerging evidence challenges this approach. A bilateral, split-body, prospective study compared suberythemogenic (70% of Minimal Erythema Dose ‒ MED) and erythemogenic (100% MED) NB-UVB regimens in dark-skinned Egyptian patients with chronic plaque psoriasis (Fitzpatrick skin types III–V). After treatment, both dosing regimens resulted in statistically similar PASI reductions and clearance rates, but the suberythemogenic side received significantly lower cumulative UVB doses (42.73 vs. 62.36 J/cm^2^, p < 0.001).^46^ These findings suggest that suberythemogenic NB-UVB protocols may offer equivalent efficacy with reduced cumulative phototoxic burden in SOC patients.

Cultural attitudes also play a critical role in phototherapy adherence. In some Asian populations, darker skin is culturally associated with manual labor and lower socioeconomic status, leading to a cultural aversion to tanning. Studies report that some Asian patients undergoing phototherapy express dissatisfaction with unwanted tanning, directly affecting adherence. These cultural nuances should inform shared decision-making during treatment planning.^44^, ^47^

In clinical practice, these findings advocate for personalized phototherapy dosing strategies in SOC patients, balancing efficacy with the minimization of adverse effects like PIH and patient-reported cosmetic concerns. Suberythemogenic NB-UVB protocols now represent an evidence-based option to optimize outcomes while minimizing pigmentary side effects in these populations.^46^

### Classical systemic therapy

Classical systemic therapies, including methotrexate, acitretin, and cyclosporine, remain foundational options for the management of moderate-to-severe psoriasis worldwide, particularly in regions where access to biologic agents is limited.

Methotrexate, a folate antagonist with immunomodulatory and anti-inflammatory effects, is widely used as a first-line systemic option due to its affordability and long-standing clinical experience. Although its efficacy appears comparable across racial and ethnic groups, there is limited representation of SOC populations in clinical trials.^6^ Hepatic safety remains a key concern, particularly in areas with higher prevalence of viral hepatitis (e.g., parts of Asia and sub-Saharan Africa), reinforcing the need for baseline screening and regular liver function monitoring.

Notably, genetic polymorphisms in the MTHFR gene (e.g., C677 T and A1298C) ‒ which affect folate metabolism and thus methotrexate pharmacokinetics ‒ occur with varying frequency across populations. Asian and Hispanic individuals, for instance, exhibit a higher prevalence of the C677T variant, which has been associated with increased toxicity risk, while African populations tend to have a lower frequency of this allele.^48^

Acitretin, a systemic retinoid, is typically reserved for patients with pustular, erythrodermic, or palmoplantar psoriasis or as a maintenance agent. It is not immunosuppressive, making it useful in patients with contraindications to immunosuppressants. However, the teratogenicity and mucocutaneous side effects can be more noticeable and distressing in SOC, particularly due to the impact on lip and facial pigmentation and potential for PIH.^14^

Cyclosporine, a calcineurin inhibitor, is fast-acting and effective in acute flares. It is commonly used for short-term control of severe disease; long-term use is limited by nephrotoxicity and hypertension, both of which may disproportionately affect certain ethnic groups. For instance, African American patients have a higher baseline risk of hypertension and may require closer monitoring when using cyclosporine.^49^ Cyclosporine’s favorable impact on disease control must be weighed against its side effect profile, especially in populations already at increased cardiovascular risk.

Importantly, there is a lack of clinical trial data specifically evaluating these therapies in SOC populations, making it difficult to draw firm conclusions about optimal dosing, efficacy, and adverse event profiles. Cultural factors may also influence treatment choice and adherence. In some cultures, there may be hesitation regarding the long-term use of systemic drugs due to fears of “chemical” medications, organ damage, or infertility ‒ factors that may not be explicitly addressed in standard consultations.^14^

## Biologic therapies for psoriasis in skin of color: current evidence and unmet needs

Biologic therapies targeting TNF-α, IL-12/23, IL-17 and IL-23 pathways have transformed the treatment landscape for moderate-to-severe psoriasis, offering high levels of skin clearance with favorable safety profiles. However, despite their widespread use, data on treatment efficacy and safety in SOC populations remain scarce and fragmented. Historically, non-white patients have been grossly underrepresented in pivotal psoriasis trials, with a recent systematic review showing that 84.3% of participants in psoriasis biologic trials were White, leaving less than 16% from all other racial and ethnic groups combined.^50^

Recent systematic reviews have tried to address this knowledge gap by pooling available data. A 2024 review, analyzing 11 phase 3 trials, included 1,393 SOC psoriasis patients, with the majority being Asian (64.3%), but Black and Hispanic patients remained strikingly underrepresented.^50^ Another review included 24 studies, again demonstrating a predominance of Asian (n = 2,740) and White (n = 8,735) participants, while only 138 Black and 728 Latino patients were enrolled across all studies.^51^

Some subgroup analyses have attempted to compare biologic responses across ethnicities, but results remain inconclusive due to low patient numbers and heterogeneity in study designs. In the review by Ferguson et al., ixekizumab showed numerically higher PASI75 response rates in Asian (98.8%) and Latino (96.6%) patients, while guselkumab appeared more effective among Black (74.2%) and White (86.8%) participants.^51^ Similarly, bimekizumab demonstrated high PASI90 and PASI100 response rates in SOC patients (85.5% and 52.6%, respectively) in Rijal et al.’s review, but again with small sample sizes (only 62 SOC patients receiving bimekizumab).^50^ Importantly, these findings are exploratory and should not yet guide clinical decision-making, given the limitations of retrospective and underpowered analyses.

Fortunately, the landscape is beginning to shift. In 2025, the publication of the VISIBLE trial (NCT05272150) marked a major milestone. This was the first large, prospective, randomized, placebo-controlled trial specifically designed to evaluate the safety and efficacy of a biologic agent (guselkumab) in SOC patients with psoriasis. The study enrolled 211 patients, all self-identifying as a race/ethnicity other than White, with over 50% having Fitzpatrick skin types IV–VI.^52^

VISIBLE had two cohorts: one focusing on moderate-to-severe body psoriasis (Cohort A) and another on scalp psoriasis, an area of particular concern in SOC patients due to its psychological and cultural relevance (Cohort B). By week 16, 59.5% of SOC patients treated with guselkumab achieved PASI90, and 41.7% reached complete clearance (PASI100). Regarding scalp involvement, 78% achieved a 90% improvement in the Psoriasis Scalp Severity Index (PSSI90), showing robust efficacy even in this challenging site.^52^

In clinical practice, the implications of the still-limited data for SOC populations suggest that, while no definitive guidelines or studies currently exist to direct the choice of a specific biologic agent based on ethnicity,^50^ intervention should not be postponed. On the contrary, establishing early and effective treatment is essential, as patients with SOC often experience a disproportionately greater impact on quality of life.^4^ Such an agile therapeutic approach is fundamental to promptly interrupt the inflammatory cascade and, consequently, mitigate the risk of post-inflammatory dyschromias, which represent a significant morbidity and a central concern for these patients.^5^

## Psoriasis management in the Brazilian Health System

In Brazil, psoriasis care is delivered through the public Unified Health System (SUS) and the private sector. According to the 2024 Brazilian Psoriasis Consensus, treat-to-target principles remain aligned with the “rule of tens” (PASI > 10, BSA > 10, or DLQI > 10) and are embedded in public and private access frameworks, helping standardize eligibility for systemic therapy and biologics nationwide.^53^

In practice, access is generally broad via SUS and regulated private coverage; however, limited specialist availability and financial constraints still impede uptake. Treatments for dyschromia, procedures and topical depigmenting agents are not routinely available in SUS and are often costly out of pocket, disproportionately affecting people in worse financial circumstances, among whom patients with higher Fitzpatrick phototypes are frequently overrepresented.

## Pustular psoriasis in skin of color

Pustular psoriasis represents a rare but severe clinical subtype of psoriasis, characterized by the presence of sterile pustules on an erythematous or inflamed background. Its most serious form, Generalized Pustular Psoriasis (GPP), is considered a dermatologic emergency, often requiring hospitalization and associated with a mortality rate of approximately 3 deaths per 100 patient-years.^54^

Epidemiological data on pustular psoriasis in SOC populations remain scarce, but existing studies suggest potential ethnic variability. A cross-sectional analysis from the University of California-San Francisco found that Asian and Hispanic/Latino patients had significantly higher odds of having pustular psoriasis compared to Caucasians (OR = 4.36 [95% CI: 1.24–17.62] for Asians; OR = 5.94 [95% CI: 1.03–31.03] for Hispanics/Latinos).^55^ Although African descent populations were underrepresented in that study, these findings suggest that non-White patients may have a relatively higher risk of pustular forms.

From a pathophysiological perspective, GPP is now recognized as an autoinflammatory condition predominantly driven by dysregulation of the IL-36 pathway, with monogenic forms linked to IL36RN mutations, especially prevalent in Asian cohorts.^54^, ^56^

Regarding treatment, historically, systemic therapies such as cyclosporine, methotrexate, and acitretin have been the mainstays for controlling acute GPP flares.^54^ Cyclosporine is often preferred for rapid control, especially in unstable cases, while acitretin plays a role in maintenance and chronic pustular variants like palmoplantar pustulosis.

Recent advances in IL-36-pathway inhibition have reshaped the management of GPP. Spesolimab, an anti-IL-36 receptor monoclonal antibody, became the first FDA-approved biologic, and is also approved by ANVISA in Brazil, specifically indicated for treating acute GPP flares in adults, based on the Effisayil-1 trial, which showed significantly faster pustule clearance and broader clinical improvement versus placebo.^56^

Importantly, most clinical trials evaluating therapies for pustular psoriasis have included very few SOC patients, limiting the ability to draw definitive conclusions about treatment efficacy and safety in these groups.

## Conclusion

Managing psoriasis in SOC presents unique challenges that extend beyond clinical features and reflect broader issues of equity, representation, and cultural competence: socioeconomic and structural barriers (including limited access to dermatologic specialists and financial constraints) disproportionately affect SOC communities and delay diagnosis and initiation of effective therapy.^15^ At the same time, implicit bias within health systems contributes to underrecognition or misdiagnosis because presentations often differ from those in lighter phototypes,^6^ and these inequities are compounded by the persistent underrepresentation of SOC individuals in clinical trials, which constrains the evidence base needed to inform guidelines and to validate the efficacy and safety of therapies across diverse populations.^57^

In Brazil, socioeconomic and structural barriers, including limited access to dermatologic specialists and financial constraints, disproportionately affect vulnerable populations, where Black and mixed-race individuals are overrepresented. These barriers, compounded by regional disparities, can delay diagnosis and initiation of effective therapy, perpetuating worse outcomes for these patients.^58^

Addressing these inequities requires culturally competent, patient-centered care with shared decision-making.^15^ Clinicians must recognize subtler SOC presentations: hyperpigmented or violaceous plaques, minimal erythema, and frequent post-inflammatory pigmentary changes.^2^

Conventional severity scoring tools like the PASI heavily weight erythema, which can be challenging to detect in darker skin tones. This reliance on erythema visualization potentially leads to the underestimation of disease severity in these patients.^5^ Therefore, there is a pressing need to develop validated, SOC-specific scoring instruments that account for differences in lesion morphology and pigmentary alterations.

Although no severity instrument has been specifically validated for SOC populations, recent studies have increasingly incorporated multimodal approaches combining clinician-reported measures, such as Investigator’s Global Assessment (IGA) and BSA, with patient-reported outcomes, including the DLQI and the Psoriasis Symptoms and Signs Diary (PSSD). In addition, tools such as the Skin Discoloration Impact Evaluation Questionnaire (SDIEQ) allow assessment of the psychosocial burden of post-inflammatory dyschromias. Together, these measures provide a more comprehensive and patient-centered evaluation of disease burden, independent of erythema visualization.^59^

Emerging technologies, including dermoscopy and artificial intelligence-based image analysis trained on diverse datasets, offer promise for future objective assessment tools,^3^, ^4^ though validation studies in SOC populations remain limited and represent a critical research priority.

Photographic documentation of psoriasis in dark skin requires refinement: standard lighting and imaging techniques often fail to capture lesion contrast in richly pigmented skin, complicating clinical monitoring and research documentation.^60^ Future tools should also incorporate assessment of residual dyspigmentation, as these sequelae are often of great concern to SOC patients and can significantly impair quality of life.^5^

Looking ahead, greater inclusion of SOC populations in clinical trials is essential to ensure that treatment recommendations are broadly applicable. Research efforts should also prioritize real-world studies and long-term outcome data reflecting diverse racial and ethnic backgrounds. Ultimately, a comprehensive, equity-focused approach is essential to deliver optimal care for all patients with psoriasis, regardless of skin color.

## ORCID ID

Ricardo Romiti: 0000-0003-0165-3831

## Financial support

None declared.

## Authors' contributions

Anderson Costa: Article writing; Data acquisition, analysis, and interpretation; critical review of the literature.

Ricardo Romiti: Study conception and design; critical review of the intellectual content; active participation in guiding the research; approval of the final version of the manuscript.

## Research data availability

The entire dataset supporting the results of this study was published in this article.

## Conflicts of interest

Anderson Costa: Consulting fees from Eli Lilly, Janssen, Novartis and Pfizer; served as a paid speaker for AbbVie, Eli Lilly, Janssen, Novartis, Pfizer, Sanofi and UCB Pharma. Ricardo Romiti: Consulting fees from AbbVie, Boehringer Ingelheim, Bristol Myers Squibb, Eli Lilly, Galderma, Janssen, LEO Pharma, Novartis, Pfizer, Sanofi, and UCB Pharma; research grants to his institution from AbbVie and has served as a paid speaker for AbbVie, Boehringer Ingelheim, BMS, Eli Lilly, Janssen, LEO Pharma, Novartis, Pfizer, Sanofi, SunPharma and UCB Pharma.
